# Methodology for Y Chromosome Capture: A complete genome sequence of  Y chromosome using flow cytometry, laser microdissection and magnetic streptavidin-beads

**DOI:** 10.1038/s41598-018-27819-x

**Published:** 2018-06-21

**Authors:** M. J. Alvarez-Cubero, O. Santiago, C. Martínez-Labarga, B. Martínez-García, R. Marrero- Díaz, A. Rubio-Roldan, A. M. Pérez-Gutiérrez, P. Carmona-Saez, J. A. Lorente, L. J. Martinez-Gonzalez

**Affiliations:** 1 GENYO (Pfizer-University of Granada-Andalusian Government Centre for Genomics and Oncological Research), Av. Ilustracion, 114 – PTS – 18016 Granada, Spain; 2 Centro di Antropologia Molecolare per lo Studio del DNA Antico, Dipartimento di Biologia, Università degli Studi di Roma“Tor Vergata”, via della Ricerca Scientifica n. 1, 00173 Rome, Italy; 3 University of Granada. Dept. of Biochemistry & Molecular Biology III - Faculty of Medicine - PTS, 18016 Granada, Spain; 4 University of Granada. Laboratory of Genetic Identification, Legal Medicine and Toxicology Department, Faculty of Medicine -PTS, 18016 Granada, Spain

## Abstract

This study is a comparison of the efficiency of three technologies used for Y chromosome capture and the next-generation sequencing (NGS) technologies applied for determining its whole sequence. Our main findings disclose that streptavidin–biotin magnetic particle-based capture methodology offers better and a deeper sequence coverage for Y chromosome capture, compared to chromosome sorting and microdissection procedures. Moreover, this methodology is less time consuming and the most selective for capturing only Y chromosomal material, in contrast with other methodologies that result in considerable background material from other, non-targeted chromosomes. NGS results compared between two platforms, NextSeq 500 and SOLID 5500xl, produce the same coverage results. This is the first study to explore a methodological comparison of Y chromosome capture and genetic analysis. Our results indicate an improved strategy for Y chromosome research with applications in several scientific fields where this chromosome plays an important role, such as forensics, medical sciences, molecular anthropology and cancer sciences.

## Introduction

The status of human Y chromosome evolution and its implications for genetics and medicine remain largely unknown. Recently published data have illustrated the need for further knowledge about the human Y chromosome, for a greater understanding of the characteristics and evolutionary forces that act on sex chromosomes, and for better tools to correctly interpret the Y chromosome’s history of long-term survival^1,2^. However there are many relevant details in the origins and functional evolution of Y chromosome when comparing Y/W genes (human/avian sex chromosomes) showing notable conservation of proto-sex chromosome expression patterns in both chromosomes^3^; or even comparing Y chromosome evolution across eight mammals identifying biases in gene content and the selective pressures that preserved the surviving ancestral genes^4^. Recent studies which compared 30 mammalian genomes reported that, despite gene gain and loss across the phylogeny, the eutherian ancestor retained a core set of 17 male-specific regions of Y chromosome genes^5^.

The Human reference genome sequence remains incomplete, as there are many satellite DNA-rich regions that continue uncharacterized^6^. Previous studies have tried to present an empirical reconstruction of human MSY (male-specific region of Y chromosome) evolution by sequencing the MSY of the rhesus macaque (*Macaca mulatta*), an Old World monkey, by comparing it to the human^7^, confirming that Y-linked genes were conserved in chimpanzees and humans, which diverged about six million years ago^8^.

The Y chromosome is one of the chromosomes with the largest proportion of repeated sequences. It has more than 40 Mb of heterochromatic regions, and the current methodological limitation of sequencing such repeated regions is the main reason why the Y chromosome has not yet been fully sequenced^9^. Short-read as well as whole genome sequencing (WGS) based long-read mapping strategies are faltering even for non-repeat Y regions (NRY), where there is no meiotic recombination map and intrachromosomal repetitive sequences are abundant^10^.

Targeted enrichment is an approach to resolve some of those difficulties. There are dozens of methods for capturing different regions of the genome. The most common methods are known as “molecular inversion probe circularization”, multiplex PCR and hybridization capture methods^11^. For a proper characterization of complex genomic regions it is important to capture and sequence large adjacent DNA fragments. To date, several authors have achieved the capture of DNA segments larger than 20 kbp in length by Region-Specific Extraction (RSE) with minimal need of a reference DNA^12^. Chromosome capture methodologies followed by sequencing offer the ability to resolve complex genomic regions.

Mitochondrial DNA (mtDNA) and the non-recombining region of the Y chromosome are the genetic markers most useful for phylogenetic and phylogeographic analyses for their specific female and male inheritance respectively. The Y-chromosome in particular, since it does not recombine, has changed over generations only through an accumulation of mutations, thereby ultimately creating patrilineal lines.

Molecular anthropology allows us to virtually travel back to the time and date of the emergence of these different mutations, thus rebuilding the migrations of the past that evolved the current set of extant mitochondrial and Y chromosome lineages with good precision^13,14^. The recent developments of Next Generation Sequencing (NGS) techniques allow us to analyze not only modern but ancient whole genomes as well, and these techniques can be used to estimate potential problems in ancient samples like contamination with modern DNA, by exploiting those molecular properties that would distinguish an endogenous human DNA from a modern exogenous contaminant^13–16^.

Due to the high interest of sequencing and capturing the Y-chromosome in several fields, such as molecular anthropology, forensics and medical genetics, specific methodologies to study Y chromosome fragments, such as haplotype-specific extraction (HSE), have been developed that allow us to discern between chromosomes from mixtures from different Y chromosomal samples^17,18^. However, there are no validated capture techniques for the whole chromosome.

It remains a challenge to consolidate an effective methodology for the reliable capture of Y chromosomal regions through procedures that are common for other parts of the genome. The isolation of certain regions of the genome - such as several genes of interest or the entire exome - are routinely done in genetic analysis. In the case of target regions with well-known nucleotide sequences, the capture is easily accomplished, due to the specificity of target sequences and the option of designing a correspondingly large number of capture probes that densely cover a large percentage of the target region for probe hybridization^19^. However, the capture of lesser-known, highly variable or more complex chromosomal regions, such as the Y chromosome, is considerably more challenging. The main reason is the difficulty of designing specific capture probes that can extract large chromosomal segments across potentially highly repetitive sequences based on a very sparse availability of unique target sequences. Moreover, the Y chromosome length and composition makes it complicated to have an efficient isolation method^20^.

A successful enrichment of the Y chromosome versus other chromosomes will allow a deeper analysis of these regions. This will improve several essential applications in phylogenetic or forensic analysis as well as in translational research. For example, obtaining an accurate phylogenetic tree with all Y chromosome variants is not possible without using Next Generation Sequencing (NGS) analysis^19,21^. Also, there is a major lack of knowledge in the genetic factors causing male infertility^22^. And finally, the haploid nature of the Y chromosome implies that any genetic alteration would immediately cause aberrant protein products for expressed genes that cannot be compensated by a second allele^1,2,22^. These questions have not been answered primarily due to the lack of adequate means for reliably enriching and studying the human Y chromosome.

## Next Generation Sequencing technologies

The development of NGS technologies has greatly expanded the field of genomics research by providing the possibility of unprecedented large-scale and high-throughput analyses. One of the most significant improvements has been focused on obtaining long range reads and bioinformatics analysis^23,24^.

Currently, NGS technologies allow for the accurate processing of a large number of samples, including a more sensitive detection of variations on a population level, the discovery of causative variants and the verification of benign single/multiple-nucleotide polymorphisms). With targeted gene capture, the relative proportion of DNA fragments originating from targeted regions is greatly increased. Moderately complex and unique genomic areas can usually be analyzed by targeted gene capture. Examples are exons, specific genes and key players in functionally important biological pathways, extended segments including exons and introns, as well as promoters or highly conserved sequences in intronic regions^25^.

An example of a commonly used NGS platform is the Illumina system, which produces up to 900 Gb of short, paired-end sequences (HiSeq. 2500: 2 × 125 bp; MiSeq: 2 × 300 bp, 13–15 Gb)^26^. More and more of reported NGS data is obtained by targeted gene capture, for example the successful confirmation of over 100 human genes implicated in syndromic and non-syndromic hearing loss^25^.

For example, among families studied through an enriched exome capture of a panel of genes involved in neuromuscular diseases, 60% had definitive diagnoses with deleterious mutations in the expected causative genes confirmed in family members. Similar diagnoses were identified in an additional 23% that had novel genotype/phenotype findings requiring functional, clinical, and/or genetic confirmation^27^. Moreover, successful genetic disease analysis through gene capture and NGS have been described for Essential tremor, Spine cerebellar ataxia, Charcot Marie Tooth disease, Friedreich ataxia, Ataxia-telangiectasia and Huntington’s disease^25^. Exome sequencing is also widely employed in clinical and research fields. Examples include the diagnosis of novel diseases and finding novel causative mutations for known disease phenotypes, used for particularly difficult-to-diagnose patients, prenatal diagnosis and early diagnosis of debilitating disease^28^. The major providers of exome capture platforms are NimbleGen, Agilent Technologies and Illumina, each having different designs and strengths^28^.

However, there is still a scarcity of data concerning *de novo* sequence analysis, which is required for a whole sequence discovery, as exemplified in the current Y chromosome application. Nowadays, an exhaustive and complete cartography of the Y chromosome is only guaranteed by the physical isolation of this chromosome followed by *de novo* sequence assembly^29^. Doing so is a complex analysis, mainly because the type of reads obtained typically do not cover all regions of interest for the study in a continuous pattern (‘sequence-coverage gaps’). Moreover, repetitive sequences and copy number sequences complicate the analysis and also prevent a contiguous sequence assembly (‘satellite-associated gaps’). The workflow, economy and read length of NGS technologies have improved, but *de novo* sequencing analysis has not been developed at the same rate. Although genome sequencing is now routine in many laboratories, translating the raw sequence data of complex and repetitive regions into an accurate and comprehensive bioinformatic assembly remains a formidable challenge^29^.

There are many current methodologies for collecting populations of whole chromosomes or chromosomal target regions for their subsequent DNA analysis. Although laser capture microdissection (LCM) is generally used to isolate specific cells from fixed tissue sections, it has also been effective in the isolation of living cells for re-culture and isolation of individual chromosomes. Preparation of chromosome paints^30^, fluorescence *in situ* hybridization (FISH)^31^ and degenerate oligonucleotide-primer PCR (DOP-PCR)^32^ combined with LCM are already described. The aim of this study was to determine the most effective protocol (in terms of cost and accurate sequence conservation) for the capture and sequence analysis of the Y chromosome. Although the Y chromosome is one of the smallest chromosomes in the human genome with a size around 60 Mb, its sequence remains partly unknown in heterochromatic regions. A better method to genetically characterize changes in the Y chromosome creates a tool that is better suited to help us understand its evolution and the genetic contributions of this variation. Successfully being able to do so is highly valuable for many applications in different scientific fields such as molecular anthropology, forensics and biomedicine. To our knowledge, this is the first comparison of several distinct technologies and protocols for the isolation and whole sequence analysis of the human Y chromosome using NGS methodologies^1,2^.

## Results

### Samples quantification

**Figure 1:**
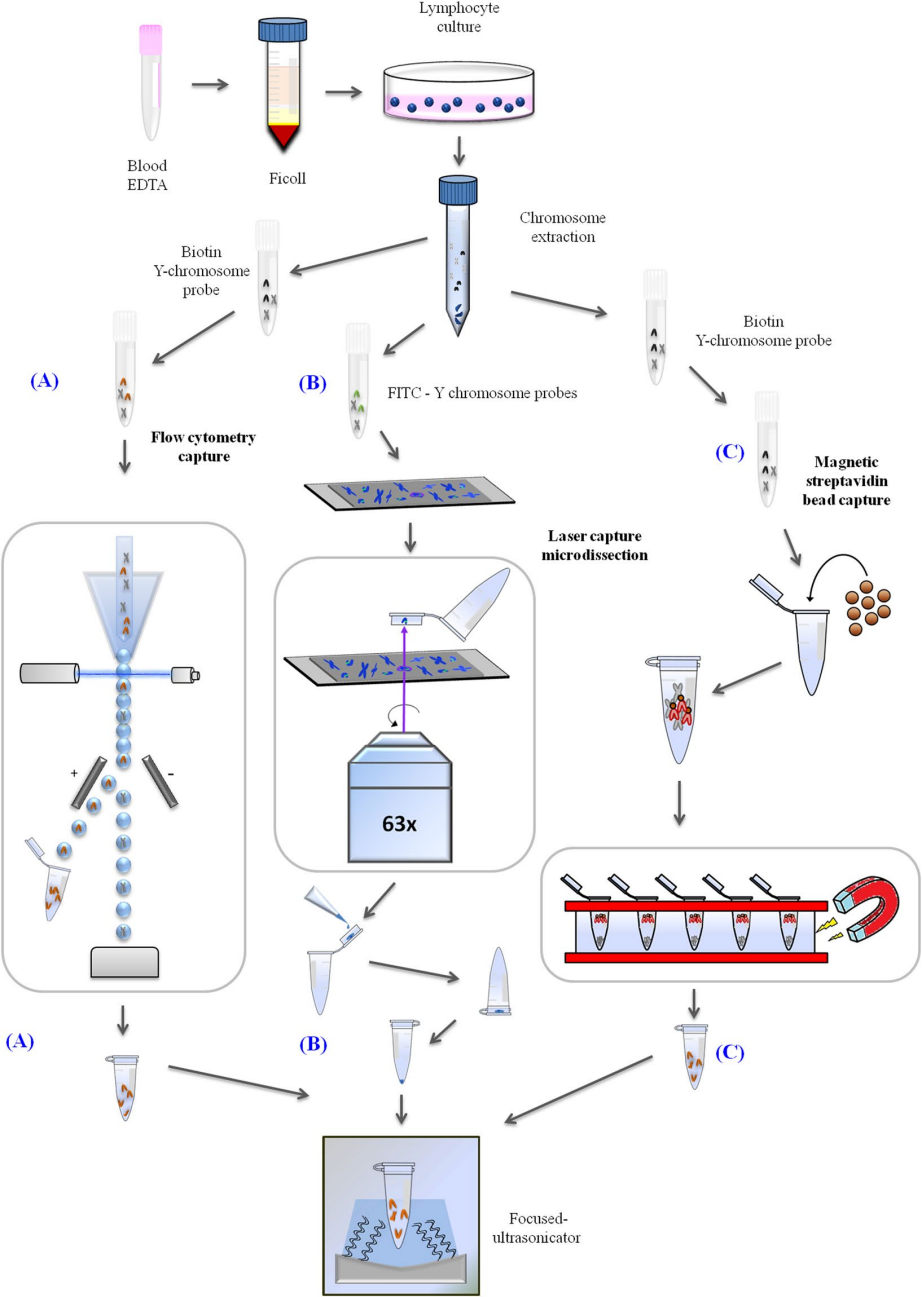
Overview of the three technologies used in this study (flow cytometry capture, laser capture microdissection, magnetic streptavidin-bead capture). For all three processes, human lymphocytes were cultured overnight, then phytohaemagglutinin (PHA) and colcemid were added to achieve a high mitotic index and accumulation of cells in metaphase. Later, metaphase chromosomes were extracted from the lymphocytes. (**A**) For flow cytometry capture, the extracted chromosomes were incubated with a specific biotin Y chromosome probe and stained with streptavidin-PE and DAPI. Y chromosomes were then sorted in a FACSAria flow cytometer. The sorted chromosomes were collected in Eppendorf (Hamburg, Germany) tubes in ddH_2_O (double-distilled water) for further processing. (**B**) For laser capture microdissection, individual chromosomes were hybridized with Y chromosome-specific probes conjugated with FITC (green), counterstained with DAPI (blue) and mounted on slides covered by polyethylene membranes. On these slides, they were selected and catapulted by the laser pressure catapulting (LPC) function in a Zeiss PALM MicroBeam IV Laser Microdissector. Y chromosomes were captured within the cap and dissolved in TE buffer. The cap was closed and the sample was spun down by centrifugation. (**C**) For magnetic streptavidin-bead capture, chromosomes were incubated with a specific biotin Y chromosome probe as in the previous procedure. Dynabeads MyOne streptavidin beads were added to the probe Y chromosome mixture and magnetic separation was performed to capture the Y chromosome on a magnetic rack. Finally, in all cases, physical fragmentation was performed before library prep and sequencing with a Covaris S2 sonicator.

After conducting the three different Y chromosome isolation processes, physical fragmentation was performed with a sonicator Covaris S2, workflow of all the samples was described in Fig. 1. Using DNA physical fragmentation, a smear or specific band was obtained, depending on the quality and quantity of the input DNA. Therefore, the input DNAs from each of the three protocols, were quantified by a Bioanalyzer 2100 (Agilent Technologies, CA, USA) using a High Sensitivity (HS) DNA Kit.

Bioanalyzer gel plots were performed by dispensing 1 µl of sample in every well of the Bioanalyzer chip. DNA samples were isolated from cell culture following the same protocol (specified in section 4.1.). The following capture protocols are explained below in section “4.2. Flow cytometry capture” (after the acquisition of a total of 500 events in 60 μl PBS), “4.3. Microdissection and image acquisition” (about 200 copies of Y chromosome were captured within the cap and dissolved in 135 µl TE (Tris-EDTA) buffer), and in section “4.4. Magnetic streptavidin-bead capture” (eluted in 160 μl of nuclease free water). They were diluted in 130 µl with Low TE Buffer in the first and second methods and concentrated to 130 µl in the third method. The three samples were sonicated using the shear program detailed in section “4.5.1. DNA Shearing”.

**Figure 2:**
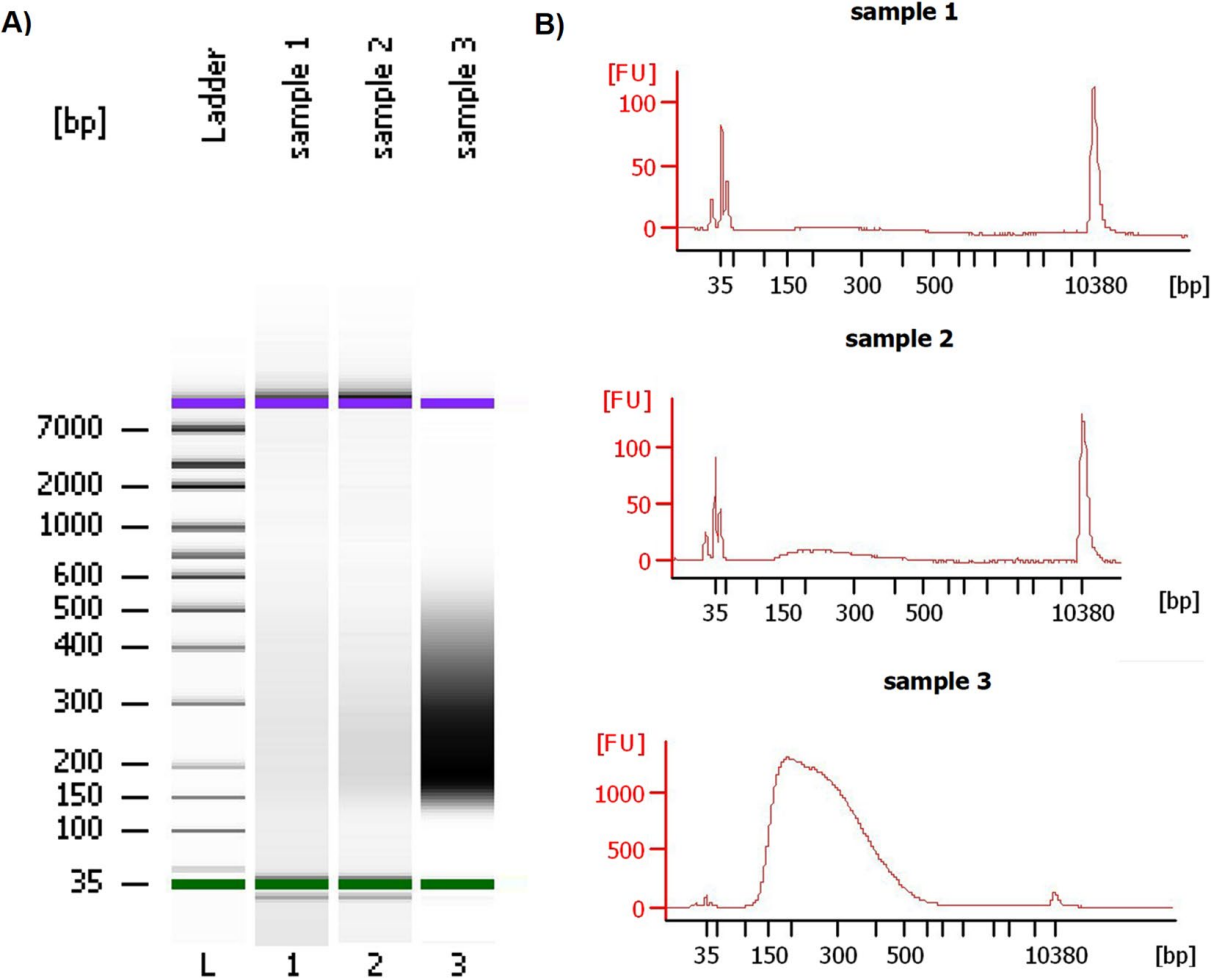
(**A**) Microfluidic electrophoretic separation of the different methods of Y chromosome capture using a DNA high sensitivity (HS) Bioanalyzer assay. Sample 1: flow cytometry capture, Sample 2: laser capture microdissection, Sample 3: magnetic streptavidin-bead capture. The HS ladder (on left) ranges from 35 base pair (bp) to 7000 bp. All sample peaks appear between the lower and upper marker peaks (35–10380 bp). (**B**) Bioanalyzer high sensitivity profiles of each capture technique. The protocol for this assay is as follows: The captured DNA (putative Y chromosome) was sonicated to a size between 150 and 500 bp and after the sonication DNA was loaded on the Bioanalyzer assay. The sonication program (see 4.5.1. DNA Shearing) was tested previously to obtain the specific fragment size, which has been verified to be proper for preparing a DNA library.

After the electrophoresis analysis, we saw that the quality and quantity of capture of the magnetic streptavidin-bead methodology was significantly better than the other processes, as can be seen in Fig. 2. The original DNA starting amount was the same in the three methods. Each sample was processed completely, adjusted to 130 μl with Low TE Buffer and sonicated (the sonication program is detailed in 4.5.1. ‘DNA Shearing’). As can be seen from the data, the specific DNA capture efficiency for the Y chromosome *versus* non-targeted chromosomes was approximately an order of magnitude greater for streptavidin-biotin-based capture compared with the other two methods.

This analysis identified the relative capture efficiencies of putative Y chromosomal DNA as provided by the three technologies, but since we did not exactly know whether this was indeed a full Y chromosome we could not determine the ratio of on- *vs*. off-target material at this point. Capture specificity and accuracy were independently determined after NGS analysis based on the results of the library sequencing data.

We performed 10 cell culture replicates with at least five million cells each per replicate. From each cell culture, we selected one for each of the three capturing methodologies (flow cytometry capture, LCM, and magnetic streptavidin-bead capture), and finally analyzed twice by NGS on Illumina and SOLID instruments (see details in the Methods section).

### NGS results

After the preparation of the libraries, quantifying them and checking their quality, they were performed multiplexed and NGS analysis by NextSeq and SOLID. We obtained the best coverage of Y chromosome reads after capture by streptavidin-biotin bead-based selection. After mapping the reads from DNA sequencing (based on DNA obtained from several capture methods), we found that the most selective methodology was bead-based capture. In contrast, reads generated via Y chromosome capture through flow cytometry capture and LCM were located all over the genome. The read depth of Y chromosome from bead-based capture was significantly higher compared to reads from all other chromosomes.

#### Illumina

Alignment quantification reads per kilobase per million: Reads per kilobase per million mapped reads formula (RPKM) is used to quantify gene expression from RNAseq experiments. It facilitates transparent comparison of alignment levels both within and between samples, eliminating the influence of different gene length and sequencing discrepancy on the calculation of gene expression. The formula for RPKM is as follows:

$${\rm{RPKM}}=\frac{{\rm{ER}}\times {10}^{9}}{{\rm{EL}}\times {\rm{MR}}}$$

where *ER* is the number of mapped reads in the gene’s exons, *EL* is the sum of the length of all exons in kilo-bases, and *MR* is the total number of mapped reads. We adapted the RPKM formula to provide a normalized measure of the number of reads that align with each chromosome based on their size: ER equals the number of mapped read in each chromosome and EL the chromosome length.

Dealing with multi-mapping reads: Multi-Mapping Reads are reads that map in more than one location. This is due to the presence of homologous or repetitive sequences throughout the genome. With the so-called -k1 option, Bowtie2 looks for multiple alignments and reports the best fit. Using -k10 option, Bowtie2 reports up to 10 valid ALNS (Alignments) per read. Multi-mapped reads have a lower MAPQ (MAPping Quality) value. Finally, we performed several strategies to improve the mapping in this chromosome.

**Figure 3:**
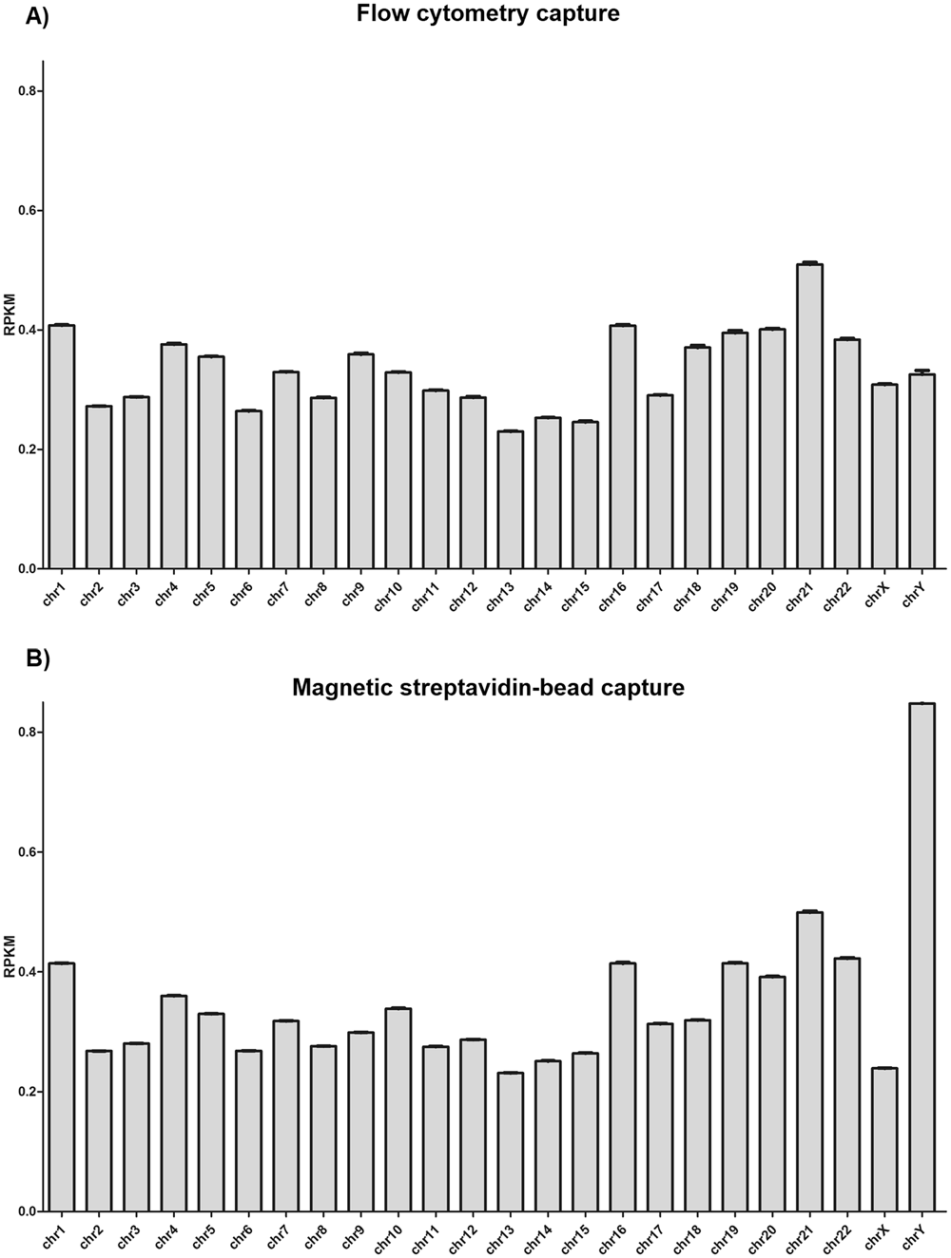
Representation of reads that map to each chromosome. (**A**) Flow cytometry capture. (**B**) Magnetic streptavidin-bead capture and collection methodology. The X-axis shows each analyzed chromosome and Y-axis shows RPKM (reads per kilobase per million mapped reads) representation.

The mapping algorithm could not assure the actual origin of the sequence when it maps in different regions, so the quality alignment value is lower than in unique mapped reads. The first strategy was a standard mapping for obtaining a valid alignment for each read and observing low quality reads mapping in the Y chromosome. These events are mainly explained by the high rate of repetitive sequences in the Y chromosome, so alignment parameters were changed to obtain 10 alignments for each read in order to obtain a higher coverage. Details of this methodology confirmed that bead-based capture was indeed the best methodological strategy for capturing Y chromosome. See details in Fig. 3.

#### Solid

**Figure 4:**
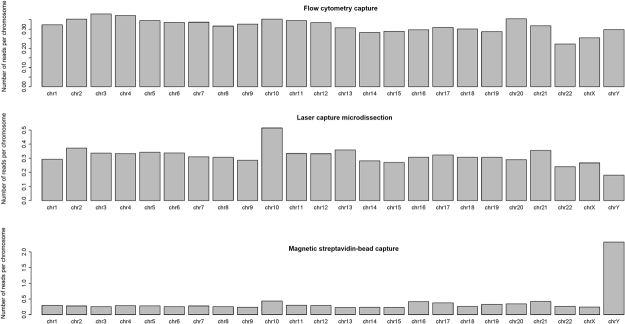
Reads that map to each chromosome. The X-axis shows the different chromosomes and the Y-axis shows the number of reads per chromosome, normalized by the chromosome length. Samtools was used to extract the number of reads that were mapped to each chromosome from bam files.

Raw data (xsq files) were obtained from the SOLiD5500xl sequencer. All raw reads were subjected to quality check, corrected and filtered using the SAET (SOLiD Accuracy Enhancement Tool) module included in the LifeScope™ version 2.5 software. Read alignment was performed with LifeScope using the default settings. Human genome assembly hg38 was used as reference. The number of mapped reads to all chromosomes was computed from bam files with Samtools and normalized with respect to the chromosome length (see Fig. 4).

## Discussion

The analysis of the whole Y chromosome genome is relevant for many applications: medicine, molecular anthropology and forensic sciences. It is known that this chromosome is one of the smallest (around 60 Mb) and the majority of the length of this chromosome (95%) is composed of “Non-Recombining Y” sequences^33^.

Currently the main challenge and difficulty of capturing and analyzing this chromosome is caused mainly by its poor content in unique genes, considering that more than 50% of its sequence is composed of repeated elements^33^. We have performed a comparative analysis of three different chromosome capture protocols to obtain an excellent tool for a whole analysis of this chromosome. We obtained the best collection results by streptavidin-bead capture (DynabeadsMyOne Streptavidin T1) (see Fig. 2) as determined by NGS analysis with both NextSeq 500 and SOLID 5500xl, which provided similar results (see Figs 3 and 4).

The streptavidin-biotin interaction has been previously cited^34^ as one of the strongest known non-covalent bonds. Thanks to this extraordinary linking strength, we could tag and manipulate big fragments or even a whole chromosome while only knowing small parts of the chromosome’s regions. This method therefore enables us to isolate an entire chromosome without the need for knowing its whole sequence. After capture and purification, the sequenced fragments can be assembled into the whole sequence of the chromosome and ideally without having significant non-targeted contributions of other chromosomes, such as repetitive sequences or regions that may be very similar to the studied chromosome but are located at other loci throughout the rest of the genome.

Our results have relevant scientific applications: the successful collection of the whole Y chromosome will offer scientists important data that were previously unknown due to its difficulty in the collection procedure. For example, it is known that infertility is one of the most increased defects in the 21^st^ century and male abnormal rates are increasing, several causes are due to Y chromosome abnormalities such as azoospermia^35^. Y chromosome analysis for instance by NGS or on arrays is one of the most promising protocols to help treat male infertility. If we can obtain more and accurate information of the Y chromosome, we can improve those analysis techniques, which will help infertility patients^36^.

In molecular anthropological analysis, the Y chromosome is currently by far the most popular marker in genetic genealogy^37^, so if we can add several new markers for genetic identification, some identification processes will be improved such as of close relatives or of ancient or highly degraded samples that today are limited to the analysis of 27 Y-STRs (short tandem repeats; 17Yfiler loci plus 10 additional Y-STRs in Yfiler Plus) (Yfiler Plus PCR Amplification Kit - Thermo Fisher Scientific, MA, USA). These Yfiler markers are not sufficient to resolve sequence ambiguities in several situations. More detail in Y chromosome genome analysis is therefore needed to improve the most accurate genetic identification of the samples.

Y-STRs analysis is the main tool to determine paternal lineages, haplotype diversity and lineage resolution across populations, in crime cases and kinship analyses, but commercial Y-STRs kits are not suitable for male individual identification because male relatives typically share the same resulting haplotype. This can also be an advantage because the non-recombining nature of male-specific Y chromosome markers allows to solve historical cases of paternity, or other types of paternal kinship dispute, as well as identification cases many generations after they occurred. Doing so is nearly impossible with recombining autosomal DNA. We hope that - aided in part by our findings - future Y-STRs kits may include more markers, particularly more RM Y-STRs (rapidly mutating Y-chromosomal short tandem repeats). Having a complete sequence of this chromosome available from many individuals is a big step for improving these kits and current male identifications, for better knowledge about the geographic distribution of Y-SNPs, and for improving the geographic resolution of paternal ancestry inference. The identification of human profiles has already been greatly improved by the addition of large numbers of multiplexed Y-STRs, as applied for autosomal STRs, Y-STRs, and SNPs such as the ForenSeq kit (Illumina, CA, USA)^38^.

Men are at a higher risk of developing and dying of sex-nonspecific cancers, but the reasons for this remains unknown. Y chromosome loss and rearrangements have been associated with different types of cancer, such as bladder cancer, male sex cord stromal tumors, lung cancer and esophageal carcinoma^33,37^. Loss of Y chromosome has been found in association with smoking, shorter survival and a higher risk of cancer (urothelial bladder cancer, pancreatic cancer, esophageal carcinoma, head and neck carcinoma, renal cell carcinoma and in cancer cell lines of hepatocellular carcinoma). It was suggested that LOY (loss of Y chromosome) in blood cells could become a predictive biomarker of male carcinogenesis^39^, because it is known that the Y chromosome is frequently lost in hematopoietic cells, representing the most common somatic alteration in men and it has a demonstrated role in cancer susceptibility^40^. So a better knowledge of more Y chromosome sequences will help us develop a deeper understanding between Y chromosome alterations and male cancer development. Mobile Elements (MEs) collectively constitute at least 51% of the human genome. There are formed by repeated sequences which show a large similarity to Y chromosome sequence. So, if we understand how these repeated sequences act in the Y chromosome we can understand better how MEs act in the whole genome. Furthermore, the Y chromosome is a strikingly hot target for human specific MEs, particularly for LTRs, which showed an insertion rate 15 times higher than the genome average^41^, however previous studies reported controversial data about LINE 1 density and total interspersed repeat elements at Y chromosome^42^.

To summarize, the full utilization of the wealth of Y chromosome genetic information has been trapped in uncharted territory to this day due to the inability of collecting and analyzing its whole genome. With the present selection of methodological success in collecting and analyzing Y chromosomes, we can greatly improve the applications of studying this chromosome in multiple scientific areas like forensics, oncology and evolution, among others.

## Methods

### Cell culture, karyotype and hybridization

For achieving a high number of chromosomes, we have performed lymphocytes cell culture collected from peripheral blood in sodium citrate anticoagulant. After being selected by Ficoll protocol we have cultured them using RPMI (Roswell Park Memorial Institute medium) 1640 medium (4 mL), fetal bovine serum (2 mL), PHA (200 µl) (Biomol, Spain) and Interleukin-2 (IL-2, 10 µl) (Affymetix, eBioscience). For chromosomes extraction, the cells should have a high mitotic index, which is difficult to achieve with primary cultures. For that reason, we increased the rate of mitosis by adding PHA^43^. When the cell count was over 5 million cells per milliliter we processed them to perform the karyotype, adding 63 µl of colcemid (10 µg/ml) (Sigma, Spain) and incubating them for 24 hours at 37 °C. The colcemid allows an accumulation of cells in metaphase^43^.

First, we perform nucleus disruption by centrifugations and incubations with hypotonic solution, other disruption methods (such as sonication and syringe sample passing) and hypotonic shock by water incubation. After this, we performed a centrifugation (10 min per 200 g) and add to the pellet 1.5 ml of frozen polyamine buffer incubating during 10 min. Finally, we performed a centrifugation for 1 min per 100 g and collected the upper phase that contains chromosomes. At this point we visualized the morphology of chromosomes in a Zeiss Axio Imager A1 epi-fluorescence upright microscope by 4′,6-Diamidino-2-Phenylindole, Dihydrochloride (DAPI) staining.

In the case of flow cytometry and magnetic streptavidin–bead capture, after 1 min centrifugation of the sample at 100 g, we collected the upper phase and incubated with 15 µl of the specific biotin Y chromosome probe (CPBR-70-316 00Y, 1125-YB, Starfish, Cambridge, UK). Afterwards, the sample with the probe was incubated for 10 min at 80 °C and hybridized 16 h at 37 °C. For flow cytometry capture, streptavidin–biotin hybridization was performed by a centrifugation (453 g for 5 min), adding of staining buffer and streptavidin-phycoerythrin (streptavidin-PE) (Affymetrix, eBioscience), followed by incubation (30 min) and wash. Magnetic streptavidin–bead capture samples were directly processed as specified in section (4.4.1) on streptavidin-biotin capture.

In the case of LCM we processed the sample with a different protocol. Y chromosome-specific probes XCE Y (centromere) and XCP Y (SRY, sex-determining region Y) (Metasystems GmbH, Altlussheim, Germany) conjugated with fluorescein isothiocyanate (FITC) were selected because they were the most specific ones to catch several regions of the Y chromosome. They both hybridize in different regions of the Y chromosome (centromere and SRY region) that was essential for performing this assay. The *SRY* gene was only found on the Y chromosome which assures the chromosomal specificity of the probes.

Finally, we performed a FISH-IS (fluorescence *in situ* hybridization in suspension) hybridization using specific green probes for Y chromosome regions (10 µl XCP Y and 10 µl XCE Y). FISH-IS was done by several centrifugations (394 g for 5 min) followed by mixing with 200-100 µl of 2 × SSC (Saline Sodium Citrate) and finally, adding 10 µl of both probes and placing the samples in thermocycler (5 min 80 °C and 2 h 37 °C). Samples were subsequently used in the LCM procedure by a use of a previously activated slide with a membrane that retains the stained chromosomes.

### Flow cytometry capture

#### Sorter capture design

**Figure 5:**
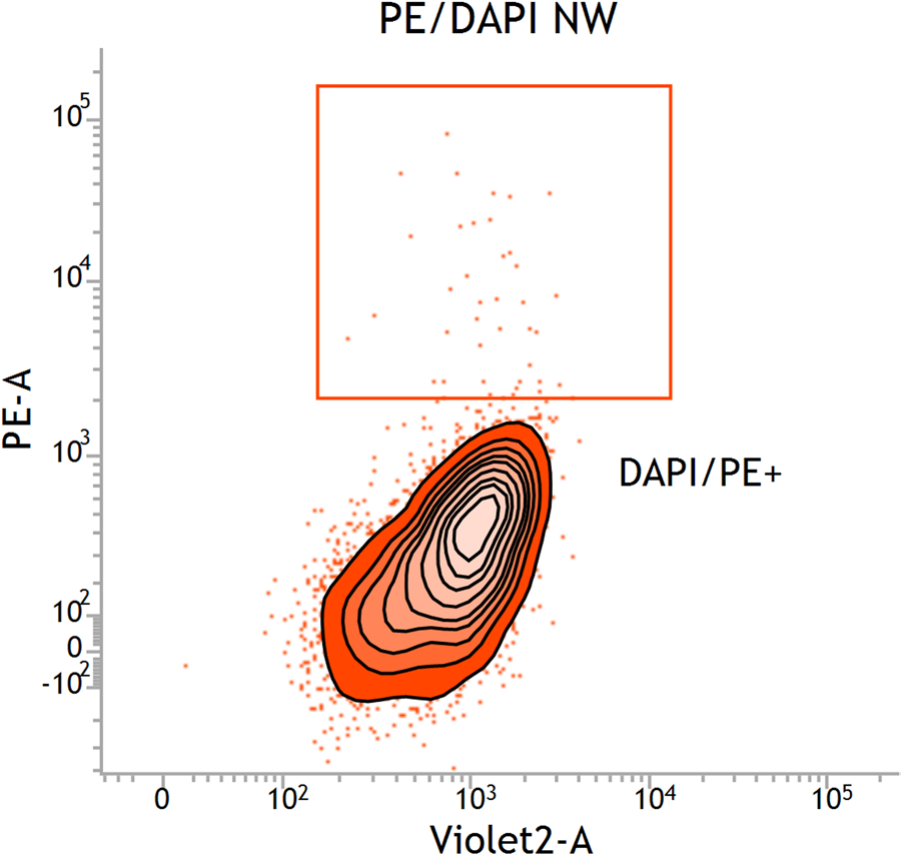
Fluorescence dot plot of stained chromosomes from the flow cytometer. The chromosomes were stained with DAPI and streptavidin-PE. The position of chromosome Y is gated.

The stained chromosome suspension was processed on a fixed-alignment benchtop high-speed cell sorter FACSAria™ equipped with low-powered, air-cooled and solid-state lasers spatially separated at the flow chamber. DAPI was excited with a Point Source Violet Solid State to emit light at 407 nm (nanometers) at 25 mW (milliwatts) laser power. DAPI fluorescence was measured through 450/40 nm band-pass filter. Phycoerythrin was excited with a Coherent Sapphire™ Solid State to emit at 488 nm at 20 mW. Phycoeythrin fluorescence was measured through 585/42 band-pass filter. Configure the FACSAria™ for high speed sorting with optimal setting of the sheath pressure to 70 psi and the drop drive frequency to 90 KHz (kilohertz) using a 70 µm nozzle tip. Select the high purity option of single cell mode. The sample flow rate was 10 µl/min. Sorted chromosomes were collected in Eppendorf tubes in ddH_2_O (double-distilled water) for further processing. For data analysis, acquire a total of 10000 events. Flow Cytometry data were acquired with FACSDiva software (BDbiosciences, USA) and results were analyzed with FACSuite software (BDbiosciences, USA). To display the data, we did a primary analysis gate set on a dual parameter dot plot comprising forward scatter (FSC; A = signal area) *versus* side scatter (SSC; W = signal weight). We try to spot the nuclei, since there will invariably be some remaining in the chromosome preparation. Using linear FSC, we can use the nuclei to help guide us to the chromosomes. Simply increase the FSC gain to reveal the chromosomes. Sorting windows were drawn on the fluorescence dot plot of DAPI (A = signal area) *versus* streptavidin-PE (A = signal area). See details in Fig. 5.

### Microdissection and image acquisition

#### Laser capture microdissection

A Zeiss PALM MicroBeam IV Laser Microdissector (Carl Zeiss, Jena, Germany) equipped with an epi-fluorescence module was used for chromosomes dissection and collection. This system consists of a 355 nm ultraviolet laser that is coupled to the light path of a Zeiss Axio Observer Z1 inverted microscope and focused through a Zeiss LD Plan Neofluar 63x/0.75 NA (numerical aperture) Korr M27 objective. The microscope stage and the laser micromanipulation procedure are computer controlled with the PALM Robo software version 4.5.

Individual chromosomes hybridized with XCE Y and XCP Y, Y chromosome-specific probes conjugated with fluorescein isothiocyanate (FITC) green dye, counterstained with DAPI and mounted on slides covered by polyethylene membrane (PEN slides; Carl Zeiss) were excited with reflected light of a HBO 100 mercury lamp and visualized with a Zeiss AxioCam ICm1 monochrome camera. In the green channel, only Y chromosomes were selected and catapulted by the laser pressure catapulting (LPC) function, with a single laser pulse and parameter of energy 63.9 µJ/pulse, directly into a Zeiss Adhesive Cap 500 opaque tube. The cap, in a tube holder, was positioned in the centre line of the laser beam above the objective. About 200 copies of Y chromosome were captured within the cap and dissolved in 135 µl TE (Tris-EDTA) buffer (Invitrogen, MA, USA). The cap was closed with the remaining tube and the sample was spun down by centrifugation.

#### Confocal laser microscopy

**Figure 6:**
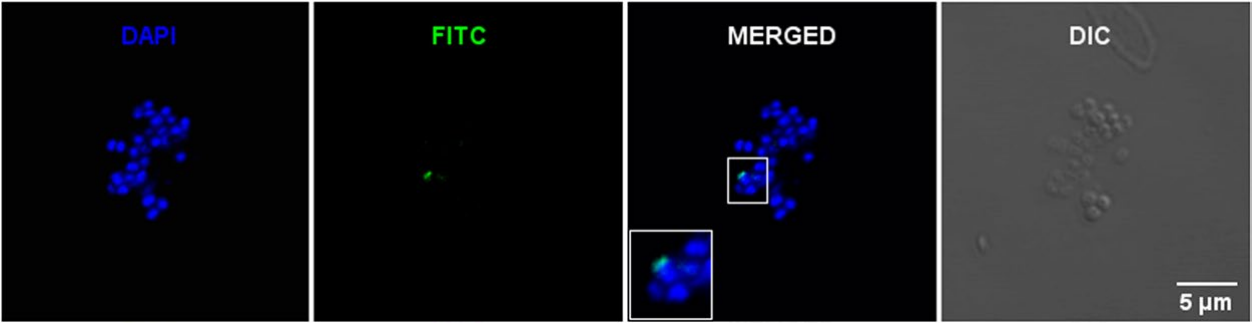
Fluorescence *in situ* hybridization (FISH) from lymphocytes was analyzed by confocal laser microscopy imaging. Representative chromosomes counterstained with DAPI (blue) and Y chromosome-specific probe conjugated with FITC (fluorescein isothiocyanate, in green) fluorescence images, their superimposition (merged image), and a transmitted light DIC (differential interference contrast) image (gray) are shown.

A Zeiss LSM 710 confocal laser scanning inverted microscope (Carl Zeiss, Jena, Germany) with Zeiss ZEN 2010 software was used for chromosome imaging. Individual chromosomes hybridized with XCE Y and XCP Y, Y chromosome-specific probes conjugated with FITC (green), and counterstained with DAPI were excited with 3% AOTF (acousto optical tunable filter) of 405 nm/30 mW diode laser line for detection of DAPI signal and 4% AOTF of 488 nm/25 mW argon laser line for green signal. A Zeiss Plan-Apochromat 63×/1.40 NA oil-immersion DIC (differential interference contrast) M27 objective and a 2.05 Airy Units pinhole were used for sequential channel acquisition of images. Details are marked in Fig. 6 that represents in blue pseudocolor (DAPI) all chromosomes; and in green pseudocolor (Y chromosome-specific probe conjugated with FITC) Y chromosomes.

### Magnetic streptavidin bead capture

#### Streptavidin–biotin-bead capture (DynabeadsMyOne Streptavidin T1)

Bead-based capture procedures were modified from related protocols (SureSelect XT Target Enrichment System for Illumina Paired-End Multiplexed Sequencing Library, Agilent Technologies).

After resuspension of the Dynabeads MyOne Streptavidin (Thermo Fisher Scientific, MA, USA), 400 µl were divided into 8 low binding plastic tubes, and washed with 200 µl of binding buffer (SureSelect Target Enrichment Box 1, Agilent Technologies), mixed on a vortex mixer for 5 s before removing the supernatant on a magnetic rack for a total of 3 washes. 15 ml of the sample previously incubated with biotinylated Y chromosome probe was concentrated for 1 h at 30 °C and separated into those 8 tubes with the Dynabeads.

The hybrid capture-bead solution was incubated on a rotator device for 80 min at room temperature. The solution was briefly spun in a centrifuge, the beads and buffer were separated on a magnetic rack and the supernatant was removed. The DNA-coated beads were then resuspended in 500 µl of SureSelect Wash 1 (SureSelect Target Enrichment Box 1, Agilent Technologies) by mixing on a vortex mixer for 5 s and briefly spun in a centrifuge, the beads and buffer were separated on a magnetic rack and the supernatant was removed. This step was repeated, incubating for 15 min at room temperature, with two mixes on a vortex mixer (this protocol step could be improved replacing vortexing by using active magnetic mixing to bring beads in contact with large, biotinylated chromosomal segments). This step was repeated, the solution was incubated for 5 min at room temperature and mixed twice on a vortex mixer. It was briefly spun in a centrifuge, the beads and buffer separated on a magnetic rack and the supernatant removed. Before the beads dried, 20 µl of nuclease-free water were added to every tube and mixed well on a vortex mixer. The samples were incubated for 2 m at room temperature and separated the beads and the supernatant on a magnetic rack. The clear supernatant was transferred from each tube to a low binding plastic tube. The 8 separated samples were pooled together in a Covaris microtube (Covaris S2, Massachusetts, USA).

### NGS Library performance

The three different methods of captured samples were kept at −20 °C until processing for NGS. We performed NGS analysis with two technologies (NextSeq 500 sequencer (Illumina, CA, USA) and SOLID 5500XL System (Applied Biosystems, CA, USA)) following a modified SureSelect XT Target Enrichment System for the preparation of libraries for Illumina Paired-End Multiplexed Sequencing and SOLiD Multiplexed Sequencing.

#### DNA Shearing

We degassed the samples in a Covaris S2 instrument for 30 min before use, chilled to 5 °C and set up the Covaris S2 for shearing as described in Table 1.

**Table 1:** Sonication program for shearing DNA in Covaris S2.

Setting	Value
Duty Cycle	10%
Intensity	5
Cycles per Burst	100
Time	6 cycles of 60 s each
Set Mode	Frequency sweeping
Temperature	4 to 7 °C

We removed the sheared DNA into a low binding plastic tube and kept it on ice. We checked the quality using the 2100 Bioanalyzer System with the Agilent Technologies High Sensitivity DNA Kit. The target peak for base pair size was approximately 150 bp. Both different SureSelect Library Prep Kits (Agilent Technologies, CA, USA) were used for preparing the mixes for enzyme reactions and PCRs.

#### End Repair

For 100 µl of the sample, we used 11 µl (10X) End Repair Buffer, 1.6 µl dNTP Mix, 1 µl T4 DNA Polymerase, 2 µl Klenow DNA Polymerase and 2.2 µl T4 Polynucleotide Kinase (enzymes, buffers and reagents, Agilent Technologies, CA, USA). This mixture was incubated for 30 min at 20 °C on a thermoblock.

#### AMPure XP bead-based purification

After maintaining room temperature for at least 30 min, the AMPure XP beads (Agencourt) were mixed thoroughly and 90 µl added to every sample, mixed again on a vortex mixer and incubated for 5 min. On a magnetic rack, the solution became clear and the supernatant was discarded. The sample was washed with two 500 µl of 70% ethanol washes, dried on the 37 °C heat block for 5 min, 15 µl of Nuclease-free Water were added, mixed on a vortex mixer, and incubated for 2 min at room temperature. We then put the tube on the magnetic stand and for 2 min. The supernatant containing the selected DNA was recovered (~15 µl).

#### Adenylation & AMPure XP bead-based purification

A mix of 11 µl of nuclease-free water, 5 µl 10X Klenow Polymerase Buffer, 1 µl dATP, 3 µl Exo (–) Klenow (enzymes, buffers and reagents from Agilent Technologies, CA, USA) were added to the sample. All were incubated for 30 min at 37 °C on a thermoblock followed by AMPure XP bead-based purification in 15 µl of nuclease-free water.

#### Ligation of paired-end adaptors & purification

A mix of 15.5 µl of nuclease-free Water, 10 µl 5X T4 DNA Ligase Buffer, 10 µl Diluted SureSelect Adaptor Oligo Mix, 1.5 µl T4 DNA Ligase (enzymes, buffers and reagents, Agilent Technologies, CA, USA) were added to the sample. The mix was incubated for 15 min at 20 °C on a thermoblock. Subsequently AMPure XP bead-based purification was carried out in 32 µl of nuclease-free water.

#### Amplification of adaptor-ligated library & purification

We used 6 µl of Nuclease-free Water, 1.25 µl SureSelect Primer, 1.25 µl SureSelect ILM Indexing Pre Capture PCR Reverse Primer, 10 µl 5X Herculase II Rxn Buffer, 0.5 µl 100 mM dNTP Mix, 1 µl Herculase II Fusion DNA Polymerase (enzymes, buffers and reagents, Agilent Technologies, CA, USA), added to the sample. The amplification conditions are listed in Table 2. Afterwards AMPure XP bead-based purification in 30 µl of nuclease-free water was performed.

**Table 2:** Incubation protocol for amplification of the adaptor-ligated library.

Cycles	Temperature	Time
1	98 °C	2 min
10	98 °C	30 s
	65 °C	30 s
	72 °C	1 min
1	72 °C	10 min
1	4 °C	∞

#### Amplification of the captured library to add index tags & purification

We used 22.5 µl of Nuclease-free Water, 10 µl (5X) Herculase II Rxn, 1 µl Herculase II Fusion DNA Polymerase, 0.5 µl 100 mM dNTP (deoxyribonucleotide triphosphate) Mix, 1 µl SureSelect ILM Indexing Post Capture Forward PCR Primer (enzymes, buffers and reagents, Agilent Technologies, CA, USA). Details of amplification conditions in Table 3. Subsequently AMPure XP bead-based purification was carried out in 30 µl of nuclease-free water.

**Table 3:** Amplification program for the captured library to add index tags.

Cycles	Temperature	Time
1	98 °C	2 min
16	98 °C	30 s
	57 °C	30 s
	72 °C	1 min
1	72 °C	10 min
1	4 °C	∞

**Figure 7:**
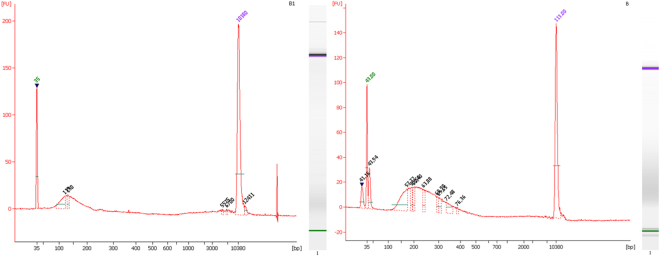
High Sensitivity DNA Kit electropherograms of during library preparation in both quality control steps: left: after shearing the DNA, and right: at the end of the library preparation. FU = fluorescence unit.

The target peak for base pair size was approximately 250 bp (Fig. 7).

### Next generation sequencing analysis

The chromosome Y captured library was quantified using Qubit fluorometric quantitation (Thermo Fisher Scientific, MA, USA), taking into account the concentration determined by Bioanalyzer for preparing the appropriate molarity.

#### NextSeq 500 (Illumina)

A dilution at 4000 pM was prepared using a solution of Tris-HCl 10 mM, pH8.5 with 0.1% Tween 20 (Bio-Rad Laboratories). It was run following the NextSeq denaturation and library dilution protocol (Illumina) in a Mid Output v2–150 cycles cartridge (Illumina) at a final concentration of 0.7 pM.

#### 5550xl SOLiD system

In the SOLiD system, once the library preparation is finished, clonal bead populations in microreactors containing template were prepared with PCR reaction components, beads, and primers using an Emulsifier system. 11 µl of a dilution of the template at 2500 pM were amplified in a plastic bag inside the Amplifier tool. After PCR, the templates were denatured and subjected to bead enrichment to separate the beads with extended templates from undesired beads. The template on the selected beads underwent a 3′ modification to allow covalent attachment to the slide.

### Sequences quality control and alignment

#### Raw data quality control

FastQC (http://www.bioinformatics.babraham.ac.uk/projects/fastqc/) is a quality control tool for high throughput sequence data. It aims to provide a simple way to do quality control checks on raw sequence data and give a quick impression of whether the data has any problems of which to be aware before doing any further analysis.

The Cutadapt tool (http://journal.embnet.org/index.php/embnetjournal/article/view/200/479) was used to trim from the raw data any large poly-N or poly-G sequences and low quality bases from the 5′ or/and 3′ ends, discarding very short reads.

#### Alignment and reference

Each sample was aligned with Bowtie2 (http://genomebiology.biomedcentral.com/articles/10.1186/gb-2009-10-3-r25) with the parameters -k1–sensitive -q -5 0 -3 0–N 0–L 22–end-to-end. Bowtie2 searches for distinct, valid alignments without mismatches for each read, where the entire read must align without trimming bases in both ends. When Bowtie2 finds a valid alignment, it continues looking for alignments that are nearly as good or better. Only the best alignment found is reported.

The samples were aligned to the last version of the Human Reference Genome GRCh38. The reference genome was downloaded from UCSC (http://hgdownload.cse.ucsc.edu/downloads.html#human). The manipulation of each alignment (sorting, indexing and calculating the reads per chromosome count) was performed by the Samtools utilities (https://github.com/samtools/samtools).

## Electronic supplementary material

Supplementary Figure 1

## References

[1] Hotaling J, Carrell DT (2014). Clinical genetic testing for male factor infertility: current applications and future directions. Andrology.

[2] Prokop JW, Deschepper CF (2015). Chromosome Y genetic variants: impact in animal models and on human disease. Physiol. Genomics.

[3] Cortez D (2014). Origins and functional evolution of Y chromosomes across mammals. Nature.

[4] Bellott DW (2014). Mammalian Y chromosomes retain widely expressed dosage-sensitive regulators. Nature.

[5] Li G (2013). Comparative analysis of mammalian Y chromosomes illuminates ancestral structure and lineage-specific evolution. Genome Res..

[6] Miga KH (2014). Centromere reference models for human chromosomes X and Y satellite arrays. Genome Res..

[7] Hughes JF (2012). Strict evolutionary conservation followed rapid gene loss on human and rhesus Y chromosomes. Nature.

[8] Hughes JF (2005). Conservation of Y-linked genes during human evolution revealed by comparative sequencing in chimpanzee. Nature.

[9] Charlesworth B (2003). The organization and evolution of the human Y chromosome. Genome Biol..

[10] Tilford CA (2001). A physical map of the human Y chromosome. Nature.

[11] .Mertes F (2011). Targeted enrichment of genomic DNA regions for next-generation sequencing. Brief. Funct. Genomics.

[12] Dapprich J (2016). The next generation of target capture technologies - large DNA fragment enrichment and sequencing determines regional genomic variation of high complexity. BMC Genomics.

[13] Jobling, M. A., Hurles, M. & Tyler-Smith, C. Human evolutionary genetics: origins, peoples &amp; disease. (Garland Science, 2004).

[14] Underhill PA (2001). The phylogeography of Y chromosome binary haplotypes and the origins of modern human populations. Ann. Hum. Genet..

[15] Sawyer S, Krause J, Guschanski K, Savolainen V, Pääbo S (2012). Temporal patterns of nucleotide misincorporations and DNA fragmentation in ancient DNA. Plos One.

[16] Stiller M (2006). Patterns of nucleotide misincorporations during enzymatic amplification and direct large-scale sequencing of ancient DNA. Proc. Natl. Acad. Sci. USA.

[17] Rothe J, Watkins NE, Nagy M (2012). New prediction model for probe specificity in an allele-specific extension reaction for haplotype-specific extraction (HSE) of Y chromosome mixtures. PLoS One.

[18] Rothe J, Nagy M (2015). Separation of Y-chromosomal haplotypes from male DNA mixtures via multiplex haplotype-specific extraction. Forensic Sci. Int. Genet..

[19] Chilamakuri CS (2014). Performance comparison of four exome capture systems for deep sequencing. BMC Genomics.

[20] Xuan J, Yu Y, Qing T, Guo L, Shi L (2013). Next-generation sequencing in the clinic: Promises and challenges. Cancer Letters.

[21] Larmuseau MHD, Van Geystelen A, Kayser M, Van Oven M, Decorte R (2015). Towards a consensus Y-chromosomal phylogeny and Y-SNP set in forensics in the next-generation sequencing era. Forensic Sci. Int. Genet..

[22] Krausz C, Escamilla AR, Chianese C (2015). Genetics of male infertility: From research to clinic. Reproduction.

[23] Diroma MA (2014). Extraction and annotation of human mitochondrial genomes from 1000 Genomes Whole Exome Sequencing data. BMC Genomics.

[24] Van Dijk EL, Auger H, Jaszczyszyn Y, Thermes C (2014). Ten years of next-generation sequencing technology. Trends in Genetics.

[25] Lin X (2012). Applications of targeted gene capture and next-generation sequencing technologies in studies of human deafness and other genetic disabilities. Hearing Research.

[26] Illumina, Inc. Illumina sequencing platforms. https://emea.illumina.com/systems/sequencing-platforms.html (2018).

[27] Tian X (2015). Expanding genotype/phenotype of neuromuscular diseases by comprehensive target capture/NGS. Neurol. Genet..

[28] Warr A (2015). Exome Sequencing: Current and Future Perspectives. G3 (Bethesda)..

[29] Chaisson MJP, Wilson RK, Eichler EE (2015). Genetic variation and the de novo assembly of human genomes. Nat. Rev. Genet..

[30] Kubickova S, Cernohorska H, Musilova P, Rubes J (2002). The use of laser microdissection for the preparation of chromosome-specific painting probes in farm animals. Chromosome Res..

[31] Langer S, Geigl JB, Gangnus R, Speicher MR (2005). Sequential application of interphase-FISH and CGH to single cells. Lab. Invest..

[32] Hobza R, Lengerova M, Cernohorska H, Rubes J, Vyskot B (2004). FAST-FISH with laser beam microdissected DOP-PCR probe distinguishes the sex chromosomes of Silene latifolia. Chromosome Res..

[33] Quintana-Murci, L. & Fellous, M. The human Y chromosome: The biological role of a ‘functional wasteland’. *Journal of Biomedicine and Biotechnology* 2001, 18–24 (2001). 10.1155/S1110724301000080 PMC79676 12488622

[34] Ozawa M, Ozawa T, Nishio M, Ueda K (2017). The role of CH/π interactions in the high affinity binding of streptavidin and biotin. J. Mol. Graph. Model..

[35] Kuroda S (2014). [Case of azoospermia patient with a chromosomal abnormality considered a ring Y chromosome]. Hinyokika Kiyo..

[36] Yuen RKC (2014). Development of a high-resolution Y-chromosome microarray for improved male infertility diagnosis. Fertil. Steril..

[37] Calafell, F. & Larmuseau, M. H. D. The Y chromosome as the most popular marker in genetic genealogy benefits interdisciplinary research. *Hum. Genet*. 10.1007/s00439-016-1740-0 (2016). 10.1007/s00439-016-1740-0 27817057

[38] Kayser M (2017). Forensic use of Y-chromosome DNA: a general overview. Hum. Genet..

[39] Noveski P (2016). Loss of Y Chromosome in Peripheral Blood of Colorectal and Prostate Cancer Patients. Plos One.

[40] Wright DJ (2017). Genetic variants associated with mosaic Y chromosome loss highlight cell cycle genes and overlap with cancer susceptibility. Nat. Genet..

[41] Tang, W., Mun, S., Joshi, A., Han, K. & Liang, P. Contribution of mobile elements to the uniqueness of human genome with more than 15,000 human-specific insertions. *bioRxiv* 83295 10.1101/083295 (2016).

[42] Skaletsky H (2003). The male-specific region of the human Y chromosome is a mosaic of discrete sequence classes. Nature.

[43] Métézeau P, Schmitz A, Frelat G (1993). Analysis and sorting of chromosomes by flow cytometry: new trends. Biol. cell.

