# Supplementary material for: Methodology for Y Chromosome Capture: A complete genome sequence of Y chromosome using flow cytometry, laser microdissection and magnetic streptavidin-beads
Source: Sci Rep. 2018 Jun 21;8:9436. doi: 10.1038/s41598-018-27819-x (PMC6013464; doi:10.1038/s41598-018-27819-x)
Supplement: Supplementary file 1 — Supplementary Figure 1 [file 41598_2018_27819_MOESM1_ESM.pdf]

TITLE PAGE

Title: METHODOLOGY FOR Y CHROMOSOME CAPTURE:

A complete genome sequence of Y chromosome using flow cytometry, laser microdissection and magnetic streptavidin-bead.

Authors: MJ Alvarez-Cubero[1,2,3] PhD*, O Santiago[1] PhD, C Martínez-Labarga[2] PhD, B Martínez-García[1] MS, R Marrero- Díaz[1] PhD, A Rubio-Roldan[1] MS, AM Pérez-Gutiérrez[1] MS, P Carmona-Saez[1] PhD, JA Lorente[1,4] MD PhD, LJ. Martinez-Gonzalez[1] PhD.

1 GENYO (Pfizer-University of Granada-Andalusian Government Centre for Genomics and Oncological Research), Av. Ilustracion, 114 – PTS – 18016 Granada, Spain

2 Centro di Antropologia Molecolare per lo Studio del DNA Antico, Dipartimento di Biologia, Università degli Studi di Roma"Tor Vergata", via della Ricerca Scientifica n. 1, 00173 Roma, Italia.

3 University of Granada. Dept. of Biochemistry & Molecular Biology III - Faculty of Medicine - PTS, 18016 Granada, Spain.

4 University of Granada. Laboratory of Genetic Identification, Legal Medicine and Toxicology Department, Faculty of Medicine -PTS, 18016 Granada, Spain.

Corresponding author (*)

Maria Jesus Alvarez Cubero. P. T. Ciencias de la Salud. Avda. de la Ilustración 114.

18016. Granada, Spain. Phone: +34 958 715 500; Fax +34 958 637 071.email: mjesusac@ugr.es.

Supplementary Figure 1. Hybridization probes localization details.

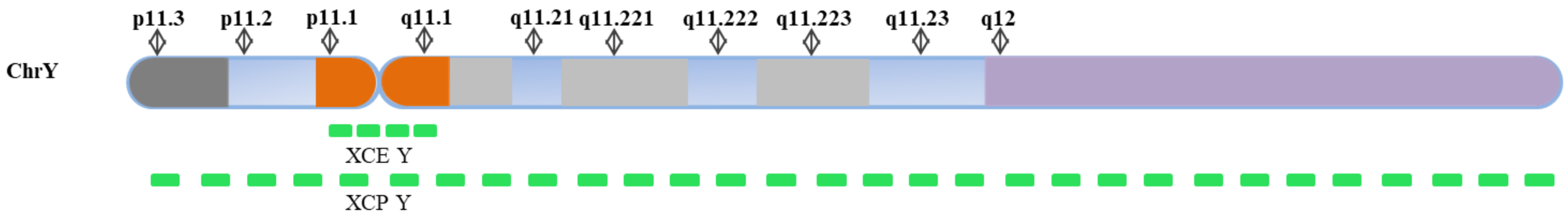

- MetaSystems Probes (Altlußheim, Germany):

XCE Y - Localization, Yp11.1-q11.1. Reference nº label green D-0824-050-FI.

XCP Y - Localization, chromosome-specific and cover the entire chromosome. Reference nº label green D-0324-050-FI.

- Cambio, Excellence in Molecular Biology (Cambridge, UK), Star*FISH©:

Localization, chromosome-specific covering the entire chromosome, shown in red. Reference nº CPBR-70-00Y.
